# AIE/ACQ Effects in Two DR/NIR Emitters: A Structural and DFT Comparative Analysis

**DOI:** 10.3390/molecules23081947

**Published:** 2018-08-04

**Authors:** Ugo Caruso, Barbara Panunzi, Rosita Diana, Simona Concilio, Lucia Sessa, Rafi Shikler, Shiran Nabha, Angela Tuzi, Stefano Piotto

**Affiliations:** 1 Department of Chemical Sciences, University of Napoli Federico II, 80126 Napoli, Italy; ugo.caruso@unina.it (U.C.); angela.tuzi@unina.it (A.T.); 2 Department of Agriculture, University of Napoli Federico II, Portici 80055 Portici NA, Italy; barbara.panunzi@unina.it; 3 Department of Industrial Engineering, University of Salerno, Fisciano 84084 SA, Italy; 4 Department of Pharmacy, University of Salerno, Fisciano 84084 SA, Italy; lucsessa@unisa.it (L.S.); piotto@unisa.it (S.P.); 5 Department of Electrical and Computer Engineering, Ben-Gurion University of the Negev, POB 653, Beer-Sheva 84105, Israel; rshikler@ee.bgu.ac.il (R.S.); nabha@post.bgu.ac.il (S.N.)

**Keywords:** DR/NIR emitter, PLQY, AIE/ACQ, DFT

## Abstract

The effects of aggregation-induced emission (AIE) and of aggregation caused quenching (ACQ) were observed and discussed on two solid materials based on a phenylenevinylene (PV) and a dicyano-PV structure. The brightest emitter in solid films shows a high fluorescence quantum yield in the deep red/near IR (DR/NIR) region (75%). The spectroscopic properties of the two crystalline solids have been described and compared in terms of crystallographic data and time dependent DFT analysis. The influence of the cyano-substituents on AIE/ACQ mechanism activation was discussed.

## 1. Introduction

The demand for optic [1,2,3,4,5] and electro-optic [6,7,8] active layers has been driven by the desire for novel technologies capable of high-yield low-cost manufacturing based on integrated optics. Fluorescent materials often represent highly required doping molecules for the active layers [9,10,11,12,13,14,15]. Organic red fluorescent dyes are typically extended π-electrons frameworks with planar conformation or a donor–acceptor type π-conjugated structure with strong intramolecular charge transfer. The development of fluorogens with deep red and/or near-IR (DR/NIR) emission is nowadays one of the hottest topics of investigation for a series of advanced technologies, i.e., in the fields of optics and electronics, bio/chemo-sensors, and bioimaging [16].

In order to emit with high quantum yields, traditional fluorescent materials are generally used as dopants. Isolated from each other (either in matrices/solutions at very low concentrations or in nanostructured guest–host systems) the well-known aggregation-caused emission quenching (ACQ), associated with the formation of less emissive species such as exciplexes and excimers, can be avoided and fluorescence maximized.

A still limited number of organic chromophores have been recently found to emit more efficiently in the aggregated state than in solution. In 2001, Tang and coworkers synthesized the first derivative almost non-emissive in solution but strongly emissive in the aggregated solid state [17]. This phenomenon was termed as aggregation-induced emission (AIE) effect. The restriction of intramolecular rotation (RIR) is by far the most frequently assumed mechanism to explain this behavior [18]. The free intramolecular rotation in solution introduces a non-radiative relaxation channel for the excited state to decay. By slowing/stopping the rotation, molecular luminescence can be restored. AIE fluorogens have recently received much research attention for their unique optical properties and extensive applications, because they are highly required for the development of efficient solid-state devices [19,20].

Many probes often show AIE effect depending on twisted structures that greatly reduce non-radiative decay channels in the solid state. Most of the AIE-active molecules reported emit blue and green lights, while the derivatives with efficient red emission in the solid state are rare. DR/NIR solid-state emitters are intrinsically narrow band gap materials typically with π-conjugated planar molecules especially vulnerable to solid-state emission quenching due to the strong π-π overlap favoring aggregation [21]. As an alternative, π-conjugated backbones such as phenylenevinylene (PV) skeleton, with strong donor (D) and acceptor (A) functionalities can show interesting DR/NIR emission in the solid state [22,23,24]. In most cases, the efficient red emission arises from an intramolecular charge-transfer (ICT) state and bulky side groups, such as cyano, can impede excessive excitonic and electronic coupling.

Herein we focus on the properties of two DR/NIR emitters based on symmetrically dinitro-substituted PV scaffolds with or without cyano-substituents in the para-position of the terminal rings, with a general acceptor-donor-acceptor (A-D-A) pattern. Their absorption/emission properties were analyzed and quantitatively evaluated recording PL quantum yield in solution and correlating PLQY measured on solid samples with X-ray diffraction analysis data and computational study results. The influence of the cyano-substituents on AIE/ACQ mechanism activation was discussed.

## 2. Results and Discussion

### 2.1. Photophysical Properties of the Fluorophores

**Scheme 1:**
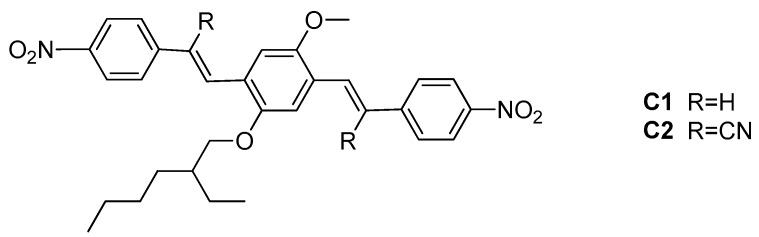
Chemical structure of the two fluorophores **C1** and **C2**. The double Knoevenagel condensation between 4-nitrophenylacetic acid or 2-(4-nitrophenyl)acetonitrile respectively and the alkoxy-terephthalaldehyde [22,25] was a convenient symmetric pattern to synthesize the 4,4’-dinitrostilbene skeleton. The identification and purity were assessed by mass spectrometry and ^1^H-NMR and by comparing melting points with the literature data [22]. The optical properties in solution were analyzed in three solvents with different polarity: dioxane < acetone<DMF (see Table 1).

The structures of the two fluorophores **C1** and **C2** are reported in Scheme 1.

**Figure 1:**
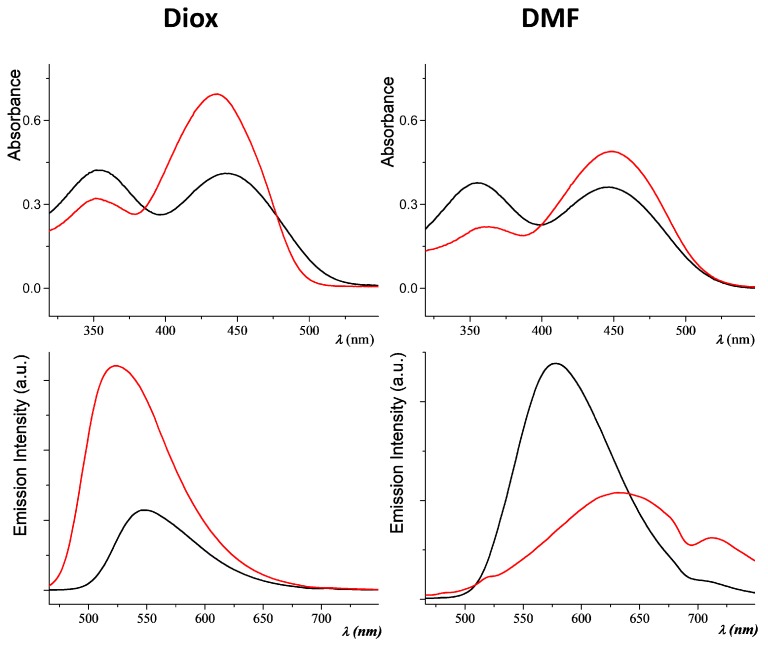
Absorption (up) and emission (down) spectra of **C1** (red line) and **C2** (black line) in dioxane (**left**) and DMF (**right**) solution.

**Figure 2:**
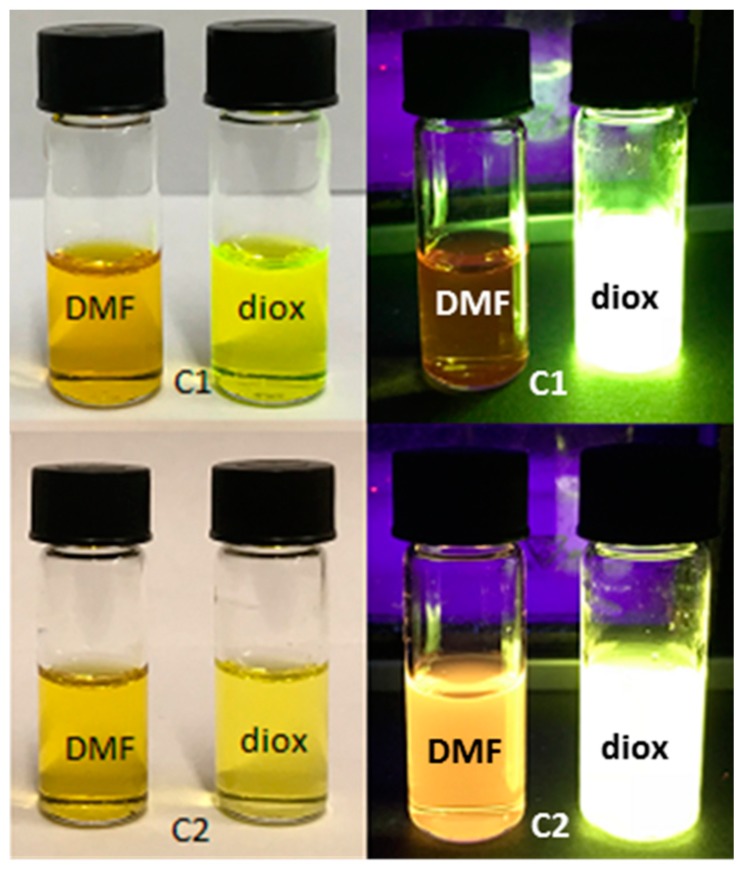
Fluorophores solutions (10%) in DMF (**left**) and dioxane (**right**) in natural (left column) and under 375 nm UV light (right column).

Both the fluorophores show a solvatochromic effect (Table 1), going from the green-yellow emission in dioxane (520–550 nm), yellow-orange in acetone (565–618 nm), to the red emission in DMF (578–620 nm). In Figure 1, the absorption and emission spectra of **C1** and **C2** can be compared in the two solvents with the higher polarity difference: dioxane and DMF. An image of the same samples observable with naked eye is shown in Figure 2. In dioxane solution, the absorption spectra show two bands separated by about 100 nm (around 350 nm and after 440 nm). The intensity of the bands are comparable for **C2**, while the second band is weaker than that of **C1**. In DMF, the two absorption bands show more similar intensity. The emission maxima are found after 520 nm for both compounds in dioxane (Figure 1).

**Figure 3:**
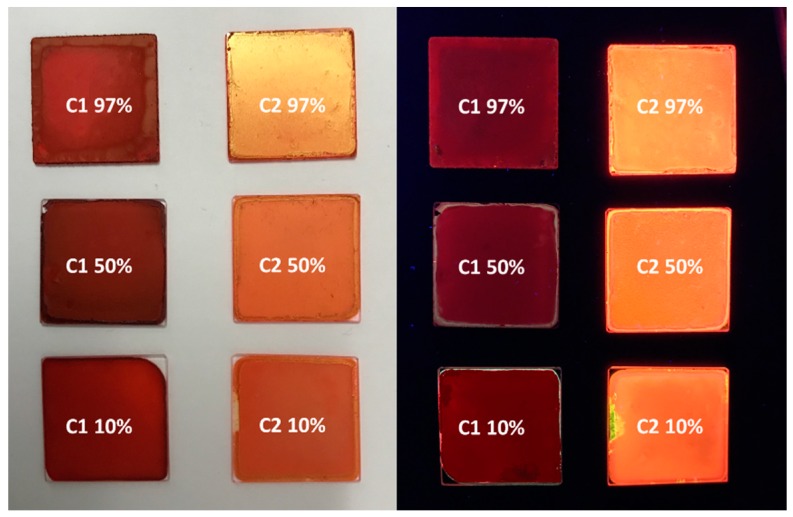
Samples of **C1** and **C2** deposed on quartz slides at different dopant percentages in natural (**left** column) and under 375 nm UV light (**right** column).

Figure 1 (bottom right) evidences the presence of two fluorescence bands. Compound **C1**, in particular, shows a NIR band peaked at 712 nm in DMF (Figure 1). As already observed [22], this is consistent with the existence of a polar excited state stabilized in a polar environment with increased probability of decaying to the ground state by non-radiative paths. The presence of a second emission band in more polar solvent is a well-known phenomenon described by Lippert [26], and it is due to an intramolecular charge transfer state stabilized by the solvent. In this case, the orientational relaxation of the solvent leads to an avoided level crossing. After relaxation, the more polar state becomes the lowest excited singlet (fluorescent) state. PL quantum yields were measured in solution by relative methods using zinc phtalocyanine as the standard [27]. PLQY for the simple PV skeleton **C1** is about twice the CN substituted derivative **C2** (see Table 1). In both cases, quantum efficiency in dioxane does not reach 35%, and considerably decreases in DMF. Optical properties were also studied in acetone/water mixture and, as already observed for similar compounds [28], the fluorescent emission is strongly quenched when small percentages of water are added (see Figure S4 of Supplementary Materials). An exhaustive quantitative analysis of the emission properties was carried out in the solid phase. From the crystalline samples, three kinds of film were prepared by spin coating. In order to test the behavior of **C1** and **C2** as dopants in solid films, we made a microdispersion of the two crystalline fluorophores in polystyrene. The samples were obtained from shattered crystals dispersed in a polystyrene (PS) matrix at 97%, 50%, or 10% by weight (Table 2). All solid compounds are light red under both natural and UV light (Figure 3). The fluorophores show broad absorption/emission bands in the solid state, sometimes resolved in separate peaks (see Table 2 and Figure S5). The samples are DR/NIR emitters, with emissions up to 620 nm for **C2**, or to 700 nm for compound **C1**. The 97% powder crystalline sample of **C1** show a second PL maximum greatly red-shifted respect to the first absorption maximum, with a remarkable Stokes Shift from 160 to 250 nm (Table 2). The measurements of PL quantum yields were performed using a 376 nm laser whose emission does not overlap with the emitted photoluminescence spectrum. The use of the crystalline fluorophores micro-dispersed in a polymer matrix usually represents a suitable way to prevent ACQ effect in the solid state [19,23]. In our case, the three polystyrene-doped films show similar PLQY values and the best result is obtained for 50% doped PS film, which is a medium level of fluorophores. For **C1**, aggregation between π-conjugated planar extended frameworks produces an ACQ effect in the solid film. In **C2**, the incorporation of the cyano substituents into the phenylenevinylene backbone suppresses the parallel stacking of aromatic rings in the solid state (see discussion below) and results to guarantee excellent PL response in the solid state of **C2** respect to **C1**. These values are among the highest values reported in literature for red emitters with similar [19,29] and not similar structure [30,31]. Fluorophore **C2** seems to be the perfect candidate to prove the AIE effect compared with the analogous non-substituted stilbene skeleton. Another easy naked eye qualitative analysis [32] was carried out in dioxane/water providing data consistent with the expected ACQ/AIE behavior of the two chromophores. The progressive addition of water (over 20%) to dioxane solutions of **C1** and of **C2** caused the precipitation and aggregation of the molecules leading to a fluorescence decrease for **C1**. On the contrary, when **C2** precipitates fluorescence increases turning to reddish color (Figure S6, Supplementary Materials).

### 2.2. X-ray Crystallography

**Figure 4:**
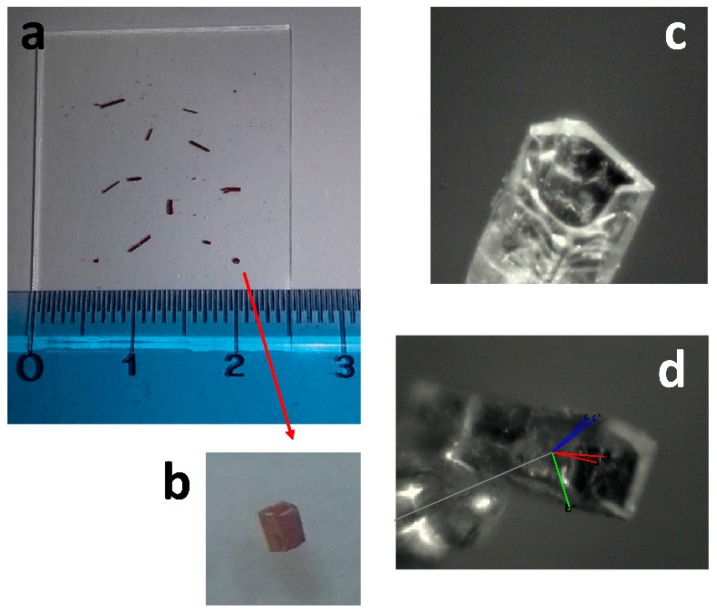
(**a**) Single rectangular cross-shaped tubular crystals of **C1** on a glass slide; (**b**) magnification 4 × of one crystal; (**c**) view of the crystal mounted on the KappaCCD diffractometer (Bruker-Nonius B.V., The Netherlands) using the KappaCCD video microscope (Bruker-Nonius B.V., The Netherlands); (**d**) unit cell axis are reported on the crystal: a or a* in red, b or b* in green, c or c* in blue.

Molecular structures of **C1** and **C2** were obtained by X-ray analysis performed on single crystals we got by slow evaporation from acetone solutions at room temperature. While it was not possible to obtain very good quality crystals of **C2**, high quality crystals of **C1** were obtained as very long, rectangular cross-shaped tubular crystals (maximum 2.5 mm elongation and 0.5 mm cross-section, see Figure 4). Such kind of topological structure in organic crystals is not frequent, and have attracted much attention because of the potential applications in optics and electronics [33].

**Figure 5:**
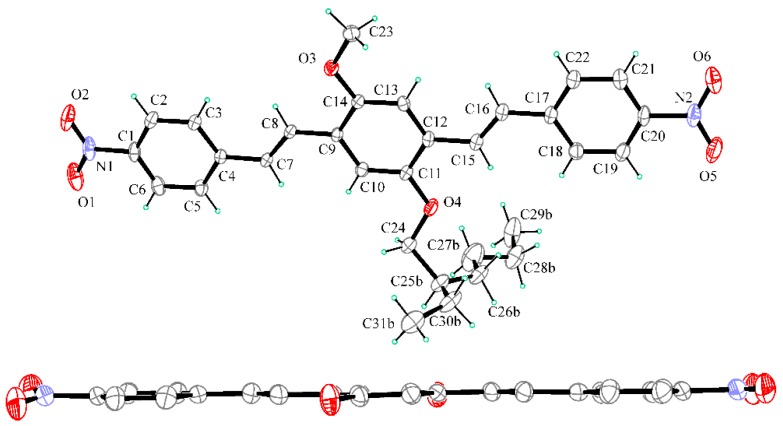
Up: Ortep view of **C1** with thermal ellipsoids drawn at 30% probability level. Only major part of the disordered aliphatic group is reported for clarity. Down: perspective view in the edge of **C9**/**C14** ring (H atoms and alkyl groups at O3 and O4 are not shown for clarity).

Compounds **C1** and **C2** crystallize in the monoclinic P2_1_/c and the triclinic P-1 space group respectively, with one molecule contained in the asymmetric unit. In both the structures, all bond lengths and angles fall within the normal range. Details of crystallographic data and refinement parameters are reported in Table S1 (Supplementary Materials). Two perspective views of **C1** molecular structure are reported in Figure 5.

**Figure 6:**
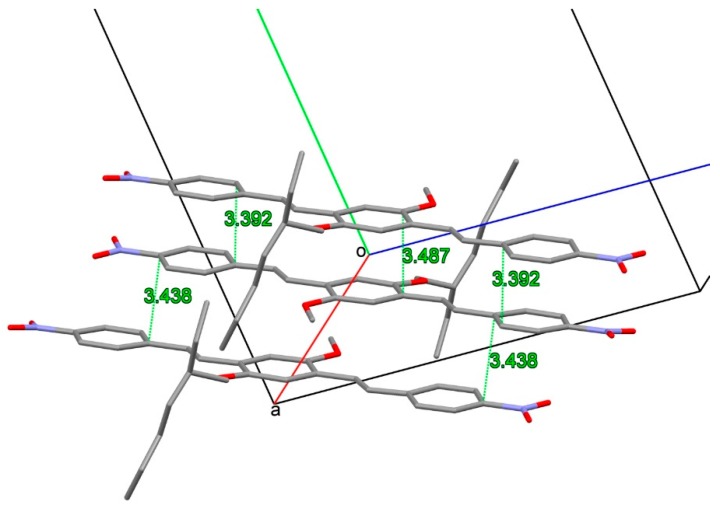
Partial packing showing the slipped stacking of molecules of **C1** in the a axis direction. Shortest distances are drawn as green dashed lines.

The molecule exhibits a completely planar conformation, with a strict coplanarity of the three aromatic rings, the two central –**C**=**C**– groups and the two terminal –NO_2_ groups. Outstanding **C1**/**C6** and **C17**/**C22** mean planes rings are 1.26(10)° and 2.51(10)° tilted with respect to the central **C9**/**C14** mean plane ring. Such geometrical peculiarity is in agreement with extended conjugation in the molecule. The branched alkyl group at O4 is disordered in two positions (refined occupancy factor 0.55/0.45) and disposes in an extended conformation quite perpendicular to the mean plane of the molecule. In the crystal packing, a herringbone pattern of molecules is observed with columns of parallel slipped molecules, stacked at about 3.4 Å across the inversion centers along a axis (Figure 6 and Figure S1, Supplementary Materials).

**Figure 7:**
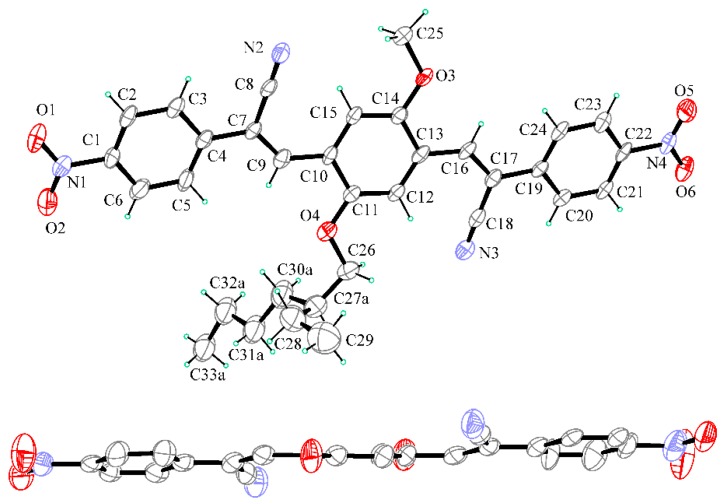
Up: Ortep view of **C2** with thermal ellipsoids drawn at 30% probability level. Only major part of disordered alifatic group attached at O4 is reported for clarity. Down: perspective view in the edge of **C10**/**C15** ring (H atoms and alkyl groups at O3 and O4 are not shown for clarity).

Two perspective views of **C2** molecular structure are reported in Figure 7. The molecule is characterized by a flat extended shape due to a fair coplanarity of the three mutually twisted aromatic rings (**C1**/**C6** and **C19**/**C24** mean planes are 13.3(2)° and 14.7(3)° tilted with respect to the central **C10**/**C15** mean plane). As usually found in similar compounds [34,35], the –NO_2_ group is coplanar with the bound phenyl ring. Similar to **C1**, also in **C2**, the alkyl group attached at O4 is disordered in two positions (refined occupancy factor 0.56/0.44) and disposes approximatively perpendicular to the mean plane of the molecule.

**Figure 8:**
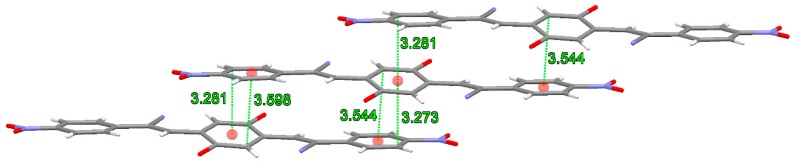
Facing of aromatic rings in **C2** packing. Shortest distances involving centroids of aromatic rings are drawn as green lines. Aliphatic groups bonded at O3 and O4 and H atoms are not reported for clarity.

In the crystal packing the –CN and –NO_2_ groups are involved in weak CH∙∙∙N and CH∙∙∙O intermolecular interactions to form layers of co-planar molecules piled up in the (a–b) direction at a strict π-stacking of 3.3 Å (Figures S2 and S3, Supplementary Materials). In the crystal, the aliphatic and aromatic parts of molecules are separated and alternate each to the other. The examination of intermolecular interactions in the crystal put in evidence some peculiar aspects that could be related to the solid-state luminescent properties. In **C2**, a small mutual torsional parallelism between adjacent molecules is found, with a slipping in the elongation axis of molecule. In this way, adjacent aromatic parts of the molecules are faced with a moderate slipping and strict π-π aromatic interactions can be undertaken (Figure 8).

**Figure 9:**
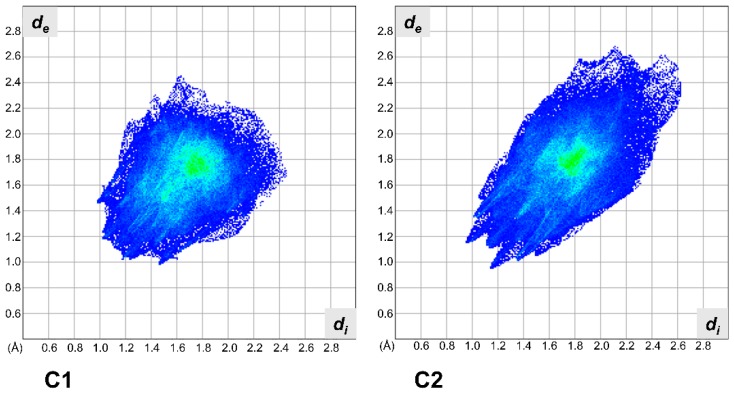
Hirshfeld fingerprint plots for **C1** (**left**) and **C2** (**right**).

This is also confirmed by the Hirshfeld surface analysis [36]. Hirshfeld surface analysis was used to show that despite packing in a nearly identical manner, **C1** and **C2** are distinctly different in their interactions and that the solid-state interactions in **C2** are dominated by the cyano moieties. The Hirshfeld analysis of **C1** shows a typical plot of an aromatic and planar molecule. The longest contact is at 4.4 Å and the closest are at around 2.2 Å. The fingerprint plot for **C2** covers a great range in both d_e_ and d_i_ (from 1.0 Å to almost 2.6 Å, in Figure 9, **C2**), and we attribute this to the presence of several anisotropic interactions, as well as the anisotropic shape of this molecule for the analysis of **C2**, which also shows the presence of several C–H···π contacts. In particular, there is an extremely short head–head H···H contact near 2.0 Å of lateral chain hydrogens. Worth of notice is the presence of extremely loose contacts at d_e_ + d_i_ = 5.1 Å on the top right region of the diagram. This is due to a strong distortion from planarity of the molecule. The distortion was induced by the presence of the cyano groups. In the Hirshfeld 2D plot the CN···H interaction is at d_e_ + d_i_ = 2.5 Å.

### 2.3. Computational Studies

**Figure 10:**
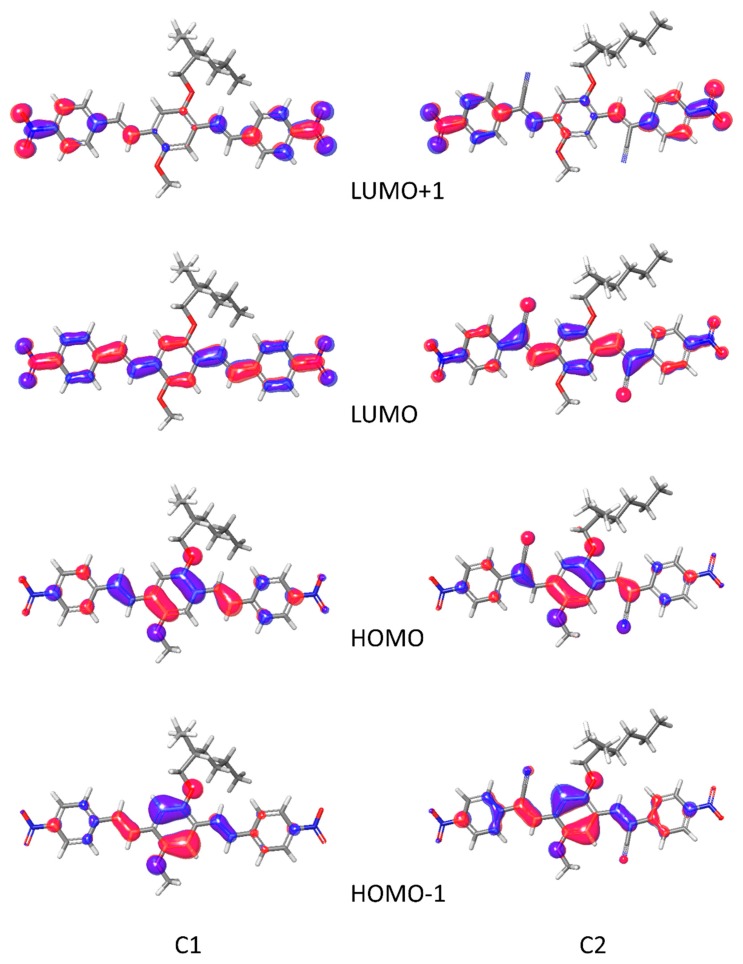
HOMO-1, HOMO, LUMO, and LUMO+1 orbitals for **C1** (**left**) and **C2** (**right**).

As experimentally demonstrated, the presence of a cyano group affects deeply the electronic properties of the material. The properties change can be due to the effect of the cyano group on the molecule itself, for example modifying the frontier orbitals or the dipole moment, or they can be due to a different packing in the crystals. Obviously, the intramolecular and the intermolecular effects are strongly correlated and, in the scientific literature, are often separated for simplicity. The geometric analysis of the crystals permitted to identify in the deviation from planarity and in the different π stack the basis for the AIE effect. We employ the time-dependent perturbation theory approach (TD-DFT) with the adiabatic local density approximation, to calculate the excitation energies. This approach has high reliability in obtaining accurate predictions for excitation energies and oscillator strengths. This analysis is necessary to clarify the role of cyano group in controlling the electronic transitions. Calculations on **C1** and **C2** in dioxane showed the localization of HOMO and LUMO orbitals (see Figure 10).

The two molecules have very similar frontier orbitals, with HOMO and LUMO delocalized overall the conjugated backbone, with only a moderate contribution of the cyano groups in **C2**. The electron withdrawing cyano groups are involved in HOMO and in LUMO. The main transitions, at 449 nm for **C1** and 477 nm for **C2**, are the HOMO → LUMO. The second main strong absorptions are due to the transition HOMO-1 → LUMO. The predicted absorption maximum is in good agreement with the experimental values (see Table 1 and Table 2), the two main absorptions corresponding to the HOMO→LUMO and HOMO-1→LUMO transitions. It is noted that, the insertion of the cyano group decreases the HOMO and the LUMO energy of **C2**, but the HOMO energy decreases not as much as the LUMO, indicating that the presence of the electro withdrawing group is beneficial to electron injection. The calculated absorption spectra are shown in Figure S7 (Supplementary Materials).

Withdrawal of the electron from a compound in a redox reaction is performed from its HOMO. The presence of the electro withdrawing nitro group in the two molecules is responsible for an oxidation potential higher than similar molecule CN-PV-NHMe [22]. **C1** and **C2** exhibits hole and electron reorganization energies (HRE and ERE respectively) that are typical for these compounds, but the presence of cyano group in **C2** increases the ERE. It is worth to notice that the reorganization energy (RE) is one of the parameters governing the hopping rate. It has long been recognized that HRE and ERE are closely related to the geometries of cation and anion states. In fact, the RE is defined as the energy difference between the charged and neutral systems at the two different geometries. The molecules with small RE possess high carrier mobility and these energies are in proportion to the deformation of the geometries in charge transfer process. In **C1** and **C2**, the HRE are smaller than the ERE indicating a smaller geometrical deformation upon electron injection compared to hole injection. The value of 0.248 eV of **C2** is remarkably small. Furthermore, as indicated in Table 3, **C1** has a minimum hole extraction potential of 6.404 eV, which demonstrates that the injection of hole into **C1** is slightly easier than in **C2**. On the contrary, **C2** has maximum electron extraction potential of 2.435 eV, indicating that the injection of electron into the **C2** becomes much easier. The above results show that the substitution of electron-withdrawing cyano group can significantly affect the electron-transporting properties of the system. In Table S2 (Supplementary Materials), a complete list of computed properties is given. In agreement with the Hirshfeld analysis, it can be seen that the presence of cyano groups can change the packing motif and increase the π–π overlap to achieve high charge mobility.

## 3. Materials and Methods

All starting products were commercially available. 4,4’-((1*E*,1’*E*)-(2-((2-ethylhexyl)oxy)-5-methoxy-1,4-phenylene) bis (ethene-2,1-diyl)) bis (nitrobenzene) (**C1**) and (2*Z*,2’*Z*)-3,3’-(2-((2-ethylhexyl)oxy)-5-methoxy-1,4-phenylene) bis (2-(4-nitrophenyl)acrylonitrile) (**C2**) were obtained respectively as described previously [22,25]. The compounds were characterized by mass spectrometry and NMR. Mass spectrometry measurements were performed using a Q-TOF premier instrument (Waters, Milford, MA, USA) equipped by an electrospray ion source and a hybrid quadrupole-time of flight analyzer. ^1^H-NMR spectra were recorded in DMSO-*d*_6_, with Bruker Avance II 400 MHz apparatus (Bruker, Leiderdorp, The Netherlands). Single crystals of **C1** and **C2** suitable for X-ray structural analysis were obtained by slow evaporation of acetone solution, at room temperature.

Optical observations were performed by using a Zeiss Axioscop polarizing microscope. UV-Visible and fluorescence spectra were recorded with JASCO F-530 (JASCO, Tokyo, Japan) and FP-750 (JASCO, Tokyo, Japan) spectrometers. Thin films were obtained by the spin coating technique of a dispersion of crystalline powder of the samples and low weight commercially available polystyrene in chloroform/hexane (2:1) by a SCS P6700 spin coater (SCS, Indeanapolis, IN, USA) operating at 600 RPM.

### 3.1. PLQY Measurements

Photoluminescence efficiency measurements on solid samples were conducted with a setup similar to that previously reported [37]. The setup consists of a 376 nm laser, whose emission does not overlap with the emitted photoluminescence spectrum, an integrating sphere (Stellarnet Inc., Tampa, FL, USA), and a photospectrometer (BLACK Comet Stellarnet Inc., Tampa, FL, USA). The setup takes into account both the exciting laser, the direct photoluminescence spectrum, and the photoluminescence that originates due to the scattering effect of the integrating sphere. The emission was measured at five different points on the sample.

### 3.2. X-ray Crystallography

Selected crystals of **C1** and **C2** were mounted in flowing N_2_ at 173 K on a Bruker Nonius Kappa CCD diffractometer, (Bruker, Karlsruhe, Germany), equipped with Oxford Cryostream apparatus (graphite monochromated MoK_α_ radiation, λ = 0.71073 Å, CCD rotation images, thick slices, ϕ and ω scans to fill asymmetric unit). Semiempirical absorption corrections (SADABS [38]) were applied. The structures were solved by direct methods (SIR97 program [39]) and anisotropically refined by the full matrix least-squares method on F^2^ against all independent measured reflections using SHELXL-2016 (2016, Sheldrick, Germany) [40] and WinGX software (2016, Farrugia, Glasgow) [41]. All the hydrogen atoms were introduced in calculated positions and refined according to the riding model with C–H distances in the range 0.95–0.99 Å and with U_iso_(H) equal to 1.2U_eq_ or 1.5U_eq_(C_methyl_) of the carrier atom. The final refinement converged to *R*_1_ = 0.0569, *wR*_2_= 0.1480 (**C1**) and *R*_1_ = 0.0987, *wR*_2_ = 0.2543 (**C2**). Both in **C1** and **C2**, the aliphatic group attached at O4 atom is disordered in two positions (refined occupancy factor 0.55/0.45 for **C1** and 0.56/0.44 for **C2**). Evidences of a complex disorder of aliphatic group in **C2** was found in difference Fourier maps but it was not possible to model the disorder in more than two positions. Some restraints were introduced on the C-C bond distances using the DFIX, SAME, and SIMU instructions of SHELXL program (2016, Sheldrick, Germany) at the last stage of refinement in **C2**. Crystal data and structure refinement details are reported in Table S1 (Supplementary Materials). The figures were generated using ORTEP-3 [41,42] and Mercury CSD 3.3 [43] programs (version 3.3, CCDC, England).

All crystal data were deposited at Cambridge Crystallographic Data Centre with assigned number CCDC 1851105 (**C1**) and 1851106 (**C2**). These data can be obtained free of charge from https://www.ccdc.cam.ac.uk/structures.

### 3.3. Computational Studies

The DFT calculations were carried out with the program Jaguar (2013, Schrödinger, LLC, NY, USA) [44] available in the Schrödinger Material Science Suite [45]. Geometry optimizations were performed with the B3LYP functional and the 6-31G** basis set. The energies of the optimized structures were estimated by additional single-point calculations using Dunning’s correlation-consistent triple-ζ basis set42 cc-pVTZ(-f) that includes a double set of polarization functions. In order to confirm proper convergence to local minima, to derive the zero-point-energy (ZPE) and entropy corrections at room temperature, we have used vibrational frequency calculation results based on analytical second derivatives at the B3LYP/6-31G**(LACVP**) level of theory.

## 4. Conclusions

We have comparatively studied two DR/NIR emitters based on phenylenevinylene PV and dicyano-PV structures (**C1** and **C2**, respectively) exhibiting fluorescent emission in the region ranging from red to NIR both solution and solid states (doped PS films). As we expected, by changing the substituents on the phenylenevinylene skeleton, e.g, by adding cyano groups, the fluorescent emission wavelength increases thanks to an aggregation induced emission (AIE) effect. In particular, while an ACQ effect was evident for **C1** in the solid, with a fluorescent quantum yields not more than 18%, a bright red emission was recorded for the fluorophore **C2**, proving a strong AIE of this fluorophore, reaching a maximum of 75% yield. Structure–property relationships were elucidated by X-ray crystallographic analysis and DFT calculations. The comparative analysis demonstrated that in the solid state, fluorescence of **C1** is quenched by the strong π-π interactions, due to the ease of aggregation between π-conjugated planar extended frameworks. In **C2**, the incorporation of the cyano substituents into the phenylenevinylene backbone suppresses close-packing in the solid state and activates the radiative channel, causing an intense aggregation-induced emission effect. Moreover, with the addition of the electron-accepting CN substituents, the energy gap (HOMO-LUMO) of the fluorophore decreases, indicating that the presence of the electro withdrawing groups is beneficial to electron injection. This analysis shows that the fluorophore **C2** can be a good candidate for application in optoelectronics, in which electron injection ability and deep red emission are highly required.

## Figures and Tables

**Table 1:** Optical data recorded in solution.

Compound	λ_abs-sol_ (nm) ^a^	λ_em-sol_ (nm) ^b^	PLQY% ^c^
**C1**	352; 437 ^i^ 357; 439 ^ii^ 361; 448 ^iii^	521 ^i^ 618; 708 ^ii^ 633; 712 ^iii^	33 ^i^ 15 ^ii^ ≤10 ^iii^
**C2**	354; 444 ^i^ 353; 440 ^ii^ 358; 446 ^iii^	550 ^i^ 565 ^ii^ 578 ^iii^	19 ^i^ 15 ^ii^ ≤10 ^iii^

^a^ UV-Visible absorbance maxima in ^i^ dioxane, ^ii^ acetone, and ^iii^ DMF solution. ^b^ Wavelength of emission maxima in ^i^ dioxane, ^ii^ acetone, and ^iii^ DMF solution. ^c^ PL quantum yield ±3% in ^i^ dioxane, ^ii^ acetone, and ^iii^ DMF solution.

**Table 2:** Optical data recorded on solid samples.

Compound% ^a^		λ_abs-film_ (nm) ^b^	λ_em-film_ (nm) ^c^	PLQY% ^d^	Stokes shift (nm) ^e^
**C1**	97%	353; 400; 505	652; 702	11 ± 0.1	252
	50%	350; 455; 500	651; 711	18 ± 0.1	196
	10%	350; 456; 559	507; 618	12 ± 0.5	162
**C2**	97%	352; 407; 567	621	66 ± 3	214
	50%	372; 475	616	75 ± 4	141
	10%	372; 494	611	67 ± 2	117

^a^ Shattered crystals of **C1** or **C2**, dispersed in PS at 97, 50 or 10% *w*/*w*. ^b^ Wavelength of UV-Visible absorbance maxima on thin film. ^c^ Wavelength of emission maxima on thin film. ^d^ PL quantum yield on thin film. ^e^ Stokes shift measured as difference between the band maxima of the absorption and emission spectra of the main electronic transition.

**Table 3:** Calculated electro-optical parameters for **C1** and **C2**.

	Hole Reorganization Energy (eV)	Electron Reorganization Energy (eV)	λ_abs_ Solution (nm)	λ_em_ Solution (nm)	Hole Extraction Potential (eV)	Electron Extraction Potential (eV)	Scaled HOMO (eV)	Scaled LUMO (eV)
**C1**	0.257	0.306	449	485	6.404	1.799	−5.877	−3.472
**C2**	0.248	0.335	477	522	6.861	2.435	−6.169	−3.980

## Supplementary Materials

The following are available online http://www.mdpi.com/1420-3049/23/8/1947/s1, Table S1: Crystallographic data and structural refinement details of **C1** and **C2**. Table S2: Calculated electro-optical parameters for **C1** and **C2**. Figure S1: Crystal packing of **C1**, viewed along a + c direction. Figure S2: Partial packing of **C2** with layer of coplanar molecules. Figure S3: View of **C2** crystal packing in the (a + 2b) direction showing layers of stacked molecules. Figure S4: Emission spectra of **C1** and **C2** in acetone/water solutions. Figure S5: Absorption and emission spectra of **C1** and **C2** in solid state. Figure S6. Fluorophores solutions in dioxane; dioxane/water 50% and dioxane/water 90% (*v*/*v*) in natural (up) and under 375 nm UV light (down). Figure S7: Predicted absorption spectra of **C1** and **C2**.
