# Supplementary material for: AIE/ACQ Effects in Two DR/NIR Emitters: A Structural and DFT Comparative Analysis
Source: Molecules. 2018 Aug 4;23(8):1947. doi: 10.3390/molecules23081947 (PMC6222639; doi:10.3390/molecules23081947)
Supplement: Supplementary file 1 [file molecules-23-01947-s001.pdf]

Supplementary Materials

AIE/ACQ effects in two DR/NIR emitters: a structural and DFT comparative analysis

Ugo Caruso [1], Barbara Panunzi [2], Rosita Diana [1,*], Simona Concilio [3,*], Lucia Sessa [4], Rafi Shikler [5], Shiran Nabha [5], Angela Tuzi [1], and Stefano Piotto [4]

[1] Department of Chemical Sciences, University of Napoli Federico II, Napoli, Italy; ugo.caruso@libero.it
[2] Department of Agriculture, University of Napoli Federico II, Portici NA, Italy; barbara.panunzi@unina.it
[3] Department of Industrial Engineering, University of Salerno, Fisciano SA, Italy; sconcilio@unisa.it
[4] Department of Pharmacy, University of Salerno, Fisciano SA, Italy; piotto@unisa.it
[5] Department of Electrical and Computer Engineering, Ben-Gurion University of the Negev, Israel; rshikler@ee.bgu.ac.il
* Correspondence: rosita.diana@libero.it; sconcilio@unisa.it; Tel.: +39-081-674-366 (R.D.); +39-089-964-115 (S.C.)

Table S1. Crystal data and structure refinement details for C1 and C2.

CCDC: 1851105 (C1) and 1851106 (C2).

	C1	C2
Empirical Formula	$C_{31}H_{34}N_2O_6$	$C_{33}H_{32}N_4O_6$
Formula weight	530.60	580.63
T (K)	173(2)	173(2)
λ (Å)	0.71073	0.71073
Crystal system	monoclinic	triclinic
Space group	P 21/c	P -1
a (Å)	8.217(2)	7.931(5)
b (Å)	22.194(4)	8.391(7)
c (Å)	16.012(3)	23.33(2)
α (°)	90.	88.93(7)
β (°)	111.93(2)	80.40(7)
γ (°)	90.	78.29(9)
V (Å^3)	2708.8(10)	1499(2)
Z	4	2
D_{calc} (Mg/m^3)	1.301	1.284
μ (mm^{-1})	0.090	0.090
F(000)	1128	612
Crystal size	0.500 x 0.250 x 0.030 mm	0.300 x 0.200 x 0.015
θ Range (°)	2.669 – 27.499	3.024 – 24.999
Refl collected / unique [R(int)]	15951 / 6040 [R(int) = 0.0349]	16770 / 5069 [R(int) = 0.1231]
Refinement method	Full-matrix least-squares on F^2	Full-matrix least-squares on F^2
Data / restraints / parameters	6040 / 0 / 421	5069 / 45 / 427
Goodness-of-fit on F^2	0.997	1.015
Final R ndices [I>2σ(I)]	R1 = 0.0569, wR2 = 0.1480	R1 = 0.0987, wR2 = 0.2543
R indices (all data)	R1 = 0.1027, wR2 = 0.1793	R1 = 0.2430, wR2 = 0.3213
$\Delta\rho_{max}$ / $\Delta\rho_{min}$ (e·A^{-3})	0.230 / -0.205	0.268 / -0.229

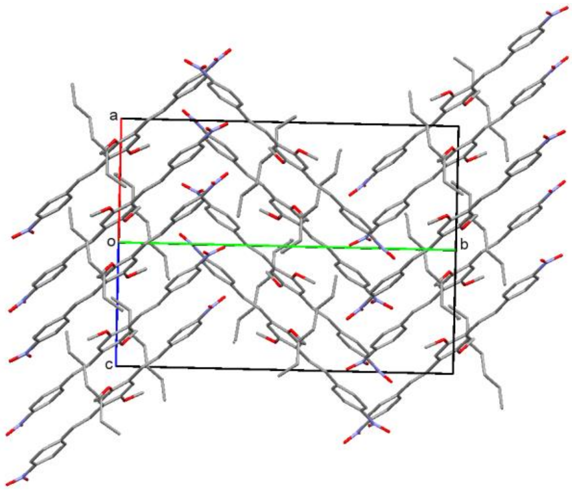

Figure S1. Crystal packing of C1, viewed along **a**+**c** direction. H atoms not shown for clarity.

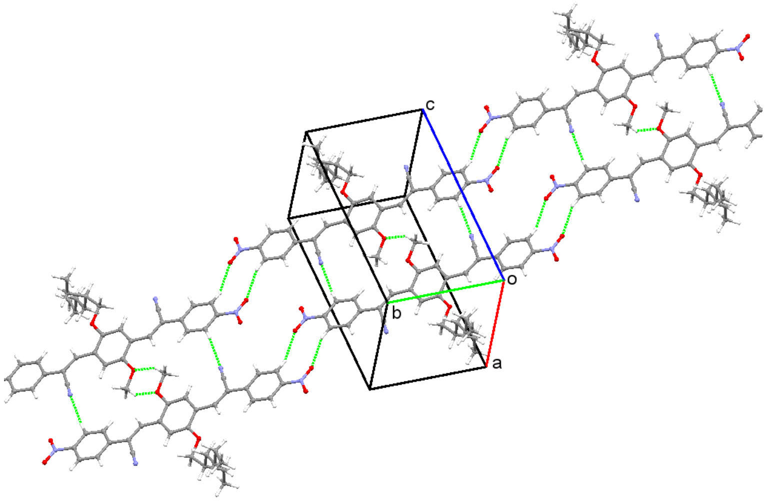

Figure S2. Partial packing of C2 with layer of coplanar molecules (intermolecular contacts CH···N and CH···O drawn as green lines)

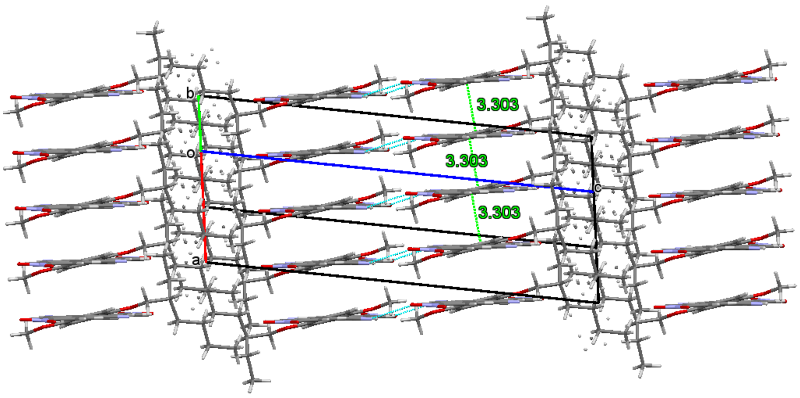

Figure S3. View of C2 crystal packing in the (**a**+2**b**) direction showing layers of stacked molecules. Weak intermolecular CH···N interactions are drawn as light blue lines; stacking distances are reported in green.

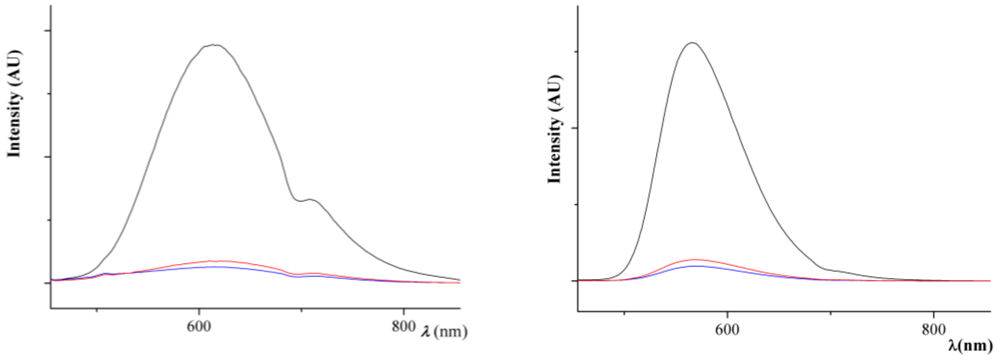

C1 **C2**

Figure S4. Emission spectra of C1 and C2 in acetone (black line) and acetone/water mixture with 5% water (red line) and 10% water (blue line).

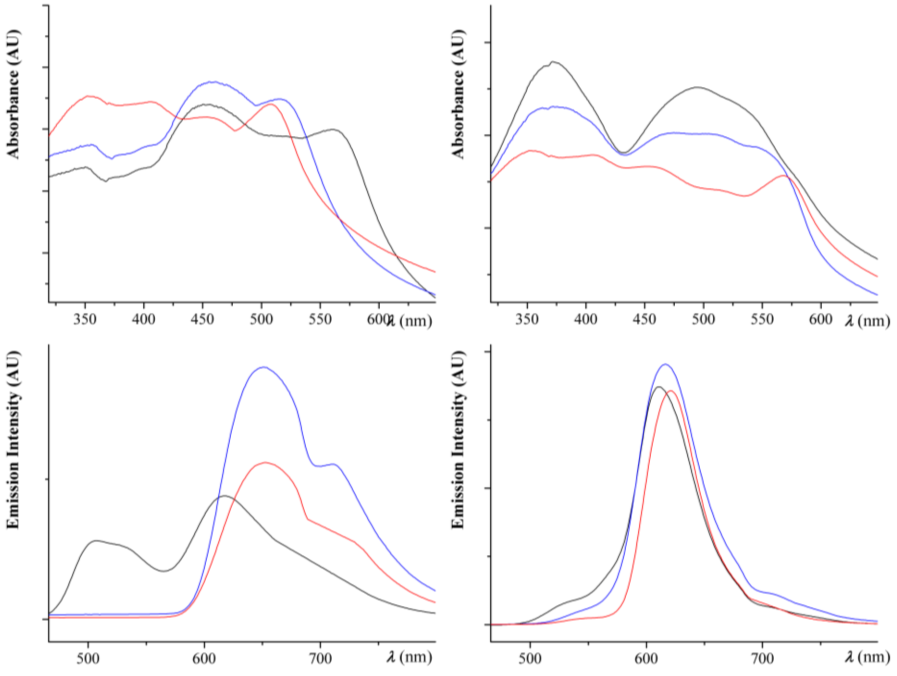

C1 **C2**

Figure S5. Absorption and emission of C1 and C2 in the solid state at different dopant percentages in polystyrene matrix: 97% (red line), 50% (blue line) and 10% (black line).

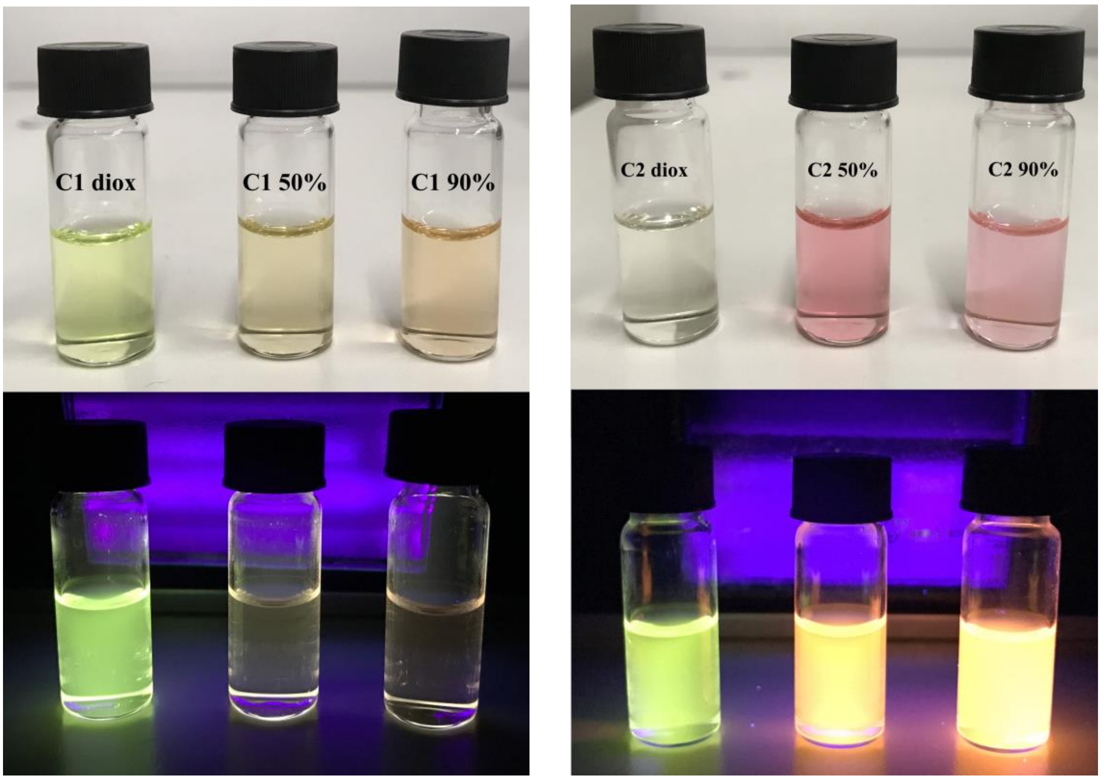

Figure S6. Fluorophores solutions (concentration 10^{-5} M) in dioxane; dioxane/water 50% and dioxane/water 90% (v/v) in natural (up) and under 375 nm UV light (down).

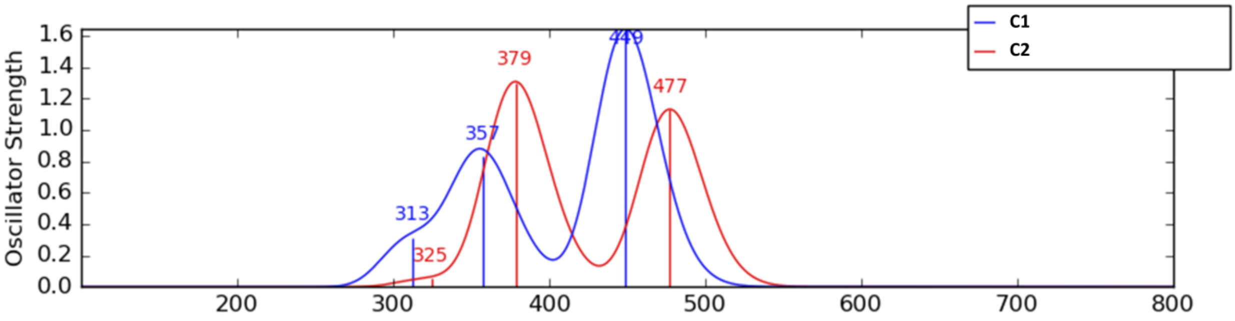

Figure S7. Predicted absorption spectra of C1 and C2

Table S2. Calculated electro-optical parameters for C1 and C2

	(eV)					(nm)		(eV)				
	Oxidation Potential	Reduction Potential	HRE [2]	ERE [2]	Triplet Energy[3]	Abs	Emiss	Triplet Stabilization Energy [3]	Hole Extraction Potential	Scaled HOMO[1]	Electron Small Polaron Stabilization Energy	T1 Vertical Absorption[3]
C1	1.347	-1.058	0.257	0.306	1.681	449	485	0.358	6.404	-5.877	0.161	1.943
C2	1.639	-0.550	0.248	0.335	1.551	477	522	0.329	6.861	-6.169	0.193	1.759

	(eV)			(Angstrom)	(eV)					(D)
	T1 Vertical Emission[3]	Triplet Reorganization Energy	Electron Extraction Potential	T1S0 RMSD [4]	S1-T2 Gap [4]	Scaled LUMO[1]	Hole Small Polaron Stabilization Energy	S1-T3 Gap[4]	S1-T1 Gap[4]	Dipole
C1	1.249	0.694	-1.799	0.187	0.223	-3.472	0.144	-0.052	1.283	0.400
C2	1.166	0.592	-2.435	0.178	0.183	-3.980	0.133	0.073	1.228	0.507

[1]Scaled HOMO and LUMO Values are calculated from the computed redox data using the energy of the NHE electrode in water, taken to be −4.28 V. [2]Hole and Electron Reorganization Energies. The reorganization energy is the sum of the energy for the neutral molecule to relax from the ion geometry to the neutral geometry and the energy for the ion to relax from the neutral geometry to the ion geometry. [3]The triplet energy is the energy of the relaxed lowest triplet state relative to the energy of the relaxed ground state. The energy of the triplet state is calculated using unrestricted DFT (UDFT) to optimize its geometry. Geometry optimizations are performed on the ground state and the triplet state, and single-point calculations for the ground state at the triplet geometry and the triplet at the ground state geometry are then performed, all in the gas phase. When the triplet reorganization energy is calculated, three other properties, T1 Vertical Absorption (eV), T1 Vertical Emission (eV), and Triplet Stabilization Energy (eV) are also calculated. [4] The energy gap between the lowest three triplet states (T1, T2, and T3) and the first excited singlet state (S1) state is calculated using TDDFT, using the S0 (ground) state as the reference.
